# Vertical Interconnection Technology for RF MicroSystem Packaging

**DOI:** 10.3390/ma17143425

**Published:** 2024-07-11

**Authors:** Yongfang Hu, Wei Sun, Yipeng Sun, Qingan Huang

**Affiliations:** 1 School of Electronic Science & Engineering, Southeast University, Nanjing 210096, China; h_yongfang@163.com (Y.H.); syp2099@163.com (Y.S.); 2 Nanjing Research Institute of Electronics Technology, Nanjing 210039, China; sunwei794527648@126.com

**Keywords:** vertical interconnection, RF microsystem, system in package

## Abstract

In this work, 1.5-level interconnections of RF microsystems with good RF performance, integration process compatibility, and high reliability are developed to meet the future demand for wireless communication microsystems in the millimeter wave band. Numerical models of 1.5-level interconnections based on solder balls with different diameters are established and analyzed using HFSS. The optimized structure parameters of 0.2 mm diameter Sn96.5Ag3Cu0.5 (SAC305) solder balls and 0.3 mm diameter Sn63Pb37 solder balls are selected for the interconnection between glass micro substrates and silicon micro substrates and between silicon micro substrates and HTCC substrates, respectively. Integration process parameters of the vertical interconnection are optimized. The micro substrate interconnection samples manufactured based on optimized integration methods and parameters show high reliability.

## 1. Introduction

Traditional packaging of RF systems is limited by their large size and cannot adapt to material changes and the shrinking size of inter-unit spacing, which are necessary to meet the future demand for miniaturized and lightweight wireless communication systems in the millimeter wave band [1,2]. To address these challenges, integrated microsystems have been developed [3]. Integrated microsystems have the advantages of small size, low power consumption, and ease of three-dimensional (3D) integration [4,5,6,7,8].

In the 3D integration of RF microsystems, multiple interconnection interfaces are involved, including those between glass micro substrates and silicon micro substrates, and between silicon substrates and high temperature co-fired ceramic (HTCC) substrates. Referring to the definitions provided by Hwang Lih Tyng et al. [9], the interconnection levels in RF microsystems are defined as follows: 1-level interconnection (interconnection between a flip chip and micro substrate), 1.5-level interconnection (interconnection between micro substrates/between micro substrates and packaging substrates), and 2-level interconnection (interconnection of external interfaces of microsystems).

To provide an electrical interconnection, there are many techniques and methods available, such as gold stud bumping [10,11,12], the Cu–Cu bonding technique [13,14,15], and the BGA soldering technique [16,17,18].

Using gold stud bumping to realize an electrical interconnection is based on thermosonic chip bond technology, which is a reliable and efficient connection technique for chip bonding [19]. However, using this technology in 1.5-level interconnections with stacked micro substrates introduces reliability risks to the microsystem. In multi-layer stacked substrates, ultrasonic energy may attenuate progressively as it penetrates through more layers, resulting in the bonding effect of the deeper substrates being less effective than that of the surface layers. Multi-layer stacking may lead to an uneven distribution of pressure across different layers, affecting the consistency and quality of the bonding. The multi-layer stacked structure may also lead to stress concentration in certain areas, especially at the interfaces between layers. Additionally, during the ultrasonic thermocompression process, the heat may accumulate at the interfaces due to differences in the thermal conductivity of materials, affecting the temperature distribution in the bonding area. All of the above can affect the reliability of gold stud bump bonding in 1.5-level interconnections.

The Cu–Cu bonding technique uses the direct bonding of copper (Cu) material to form electrical interconnections. Compared to traditional solder connections, Cu–Cu bonding can provide higher interconnection density and lower thermal expansion coefficients, meeting the needs of high-performance computing and artificial intelligence applications. However, it also has some disadvantages and challenges. Some potential drawbacks are as follows: (a) Oxidation issues: Copper is prone to oxidation at high temperatures, which can affect the quality and reliability of the bonding. Surface oxidation is a problem that needs to be overcome when forming a stable joint. (b) Process complexity: Cu–Cu bonding may require specific surface treatments or the use of capping metal layers to protect copper from oxidation, adding complexity to the process. (c) Temperature and time requirements: Although Cu–Cu bonding can be achieved at lower temperatures, in some cases, optimized bonding conditions may be needed to achieve a faster bonding process, which is especially important for mass production. Currently, Cu–Cu bonding technology is mainly used to achieve chip-to-chip connections in microelectronic packaging and three-dimensional integrated circuits (3D-ICs).

The BGA soldering technique is a self-aligned processing technology which has been widely used in vertical interconnections. BGA technology is known for its high-density interconnections, which are beneficial for microsystems that require compact and efficient use of space. The soldering process involved in BGA can provide strong and reliable electrical connections, which is crucial for the performance and durability of RF microsystems. However, when the pitch between BGA pads shrinks, the risk of short circuits increases during BGA soldering. The pad pitch is usually above 0.13 mm when the BGA soldering technique is applied in vertical interconnections. Consequently, the BGA soldering technique is primarily used in 1.5-level interconnections and 2-level interconnections.

This article primarily investigates 1.5-level interconnections in the RF microsystem integration. Several challenges are associated with 1.5-level interconnections, including the RF performance of the vertical interconnection structures, compatibility with the system integration process, and the reliability of vertical interconnections. To address these challenges, this work focuses on vertical interconnection media, vertical interconnection processes, and vertical interconnection reliability.

## 2. Materials and Methods

In the study of 1.5-level interconnections, the following three factors must be comprehensively considered.

The first is integration process compatibility. The overall integration of RF microsystems [20] involves multiple processes and various process temperatures. When designing 1.5-level interconnections, temperature gradients must be comprehensively considered as the interconnections must withstand the influence of subsequent process temperatures without affecting the reliability of chips, front solder joints, etc.

The second is under bump metallurgy (UBM). Low-loss transmission of microwave radio frequency signals typically requires a substrate metallized coating with low resistivity and a thickness exceeding 2 μm. The design of UBM layers with a thickness of over 2 μm needs to be compatible with the preset process and welding process of interconnection bumps while meeting the long-term reliability requirements of interconnection solder joints [21]. Therefore, the compatibility design of UBM structures is necessary.

The third is interconnection bumps. The insertion loss of interconnection structures at high frequency and electromagnetic compatibility issues should be fully considered during the design of the vertical interconnections between micro substrates. In vertical interconnections, coaxial structures are typically used with a signal transmission interconnection bump as the center and grounding bumps arranged at equal intervals around it. The key impedance matching structural parameters of the transmission structure include the bump diameter, the bump height, and the pad diameter [22]. The deviation between the convex point and the pad radius, as well as the change in the height of the convex point, has a significant impact on the RF transmission characteristics [23]. In the signal radiation layer, if a resonant cavity design is adopted, the geometric parameters, such as the height and diameter of the interconnection protrusions between micro substrates, have a serious influence on the transceiver performance of the RF microsystem [24]. When interconnections are made between micro substrates with different materials, the thermal expansion coefficient (CTE) mismatch of the materials should be considered [25].

### 2.1. Simulation

**Figure 1:**
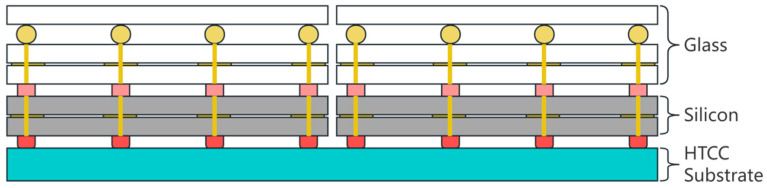
The RF microsystem structure.

The RF microsystem structure is shown as Figure 1. For the study of 1.5-level interconnections in RF microsystems, the vertical interconnections among glass micro substrates, silicon micro substrates, and HTCC substrates were investigated.

In the RF microsystem, a resonant cavity is formed by stacking glass micro substrates. The accuracy of the resonator length is the key factor in the performance of the RF system. This poses a significant challenge to the vertical interconnection solder joints between glass micro substrates. In the vertical interconnection between glass radiation units and silicon T/R modules, as well as between silicon T/R modules and HTCC substrates, substrate warping caused by multiple stacking, CTE differences between materials, and microwave insertion losses of vertical interconnection structures at high frequency are important considerations.

**Figure 2:**
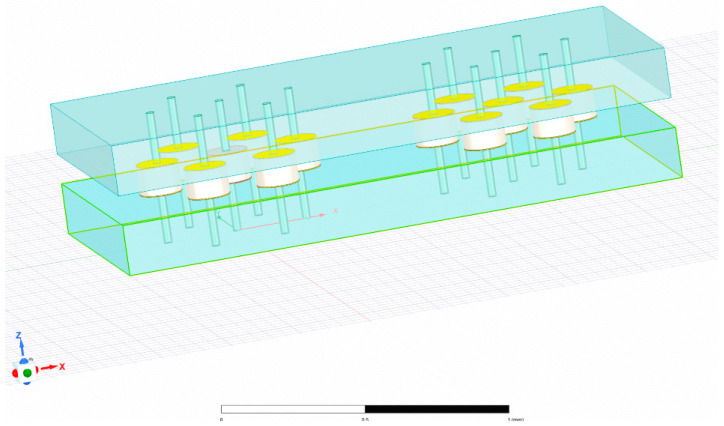
Numerical models of 1.5-level interconnections were established using HFSS.

HFSS was used to simulate the 1.5-level interconnections based on solder balls. The simulated vertical interconnection structure is shown in Figure 2 The material properties of the simulated structure are listed in Table 1.

**Figure 3:**
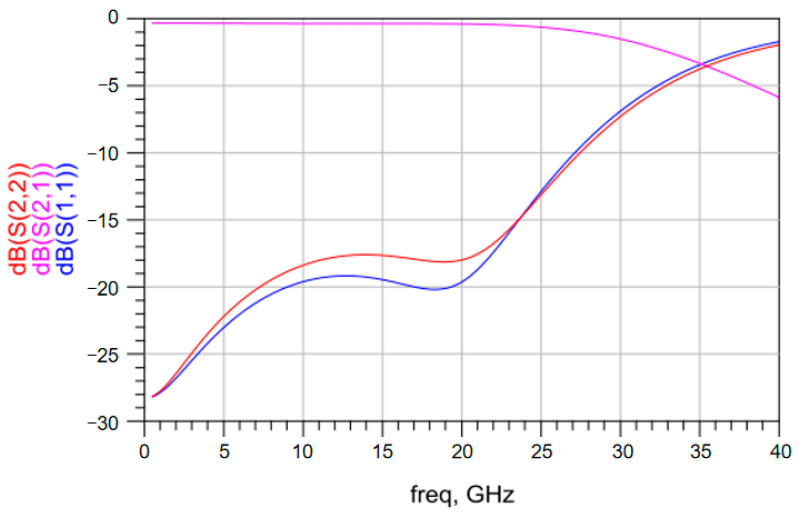
S parameters of the vertical interconnection structure based on solder balls with a diameter of 0.5 mm.

**Figure 4:**
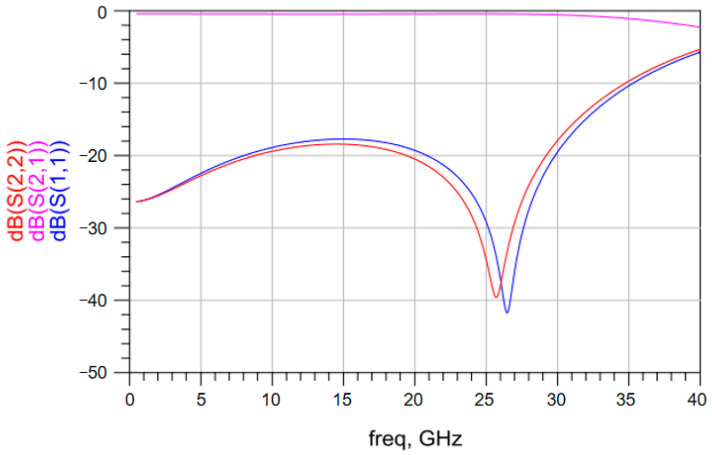
S parameters of the vertical interconnection structure based on solder balls with a diameter of 0.3 mm.

**Figure 5:**
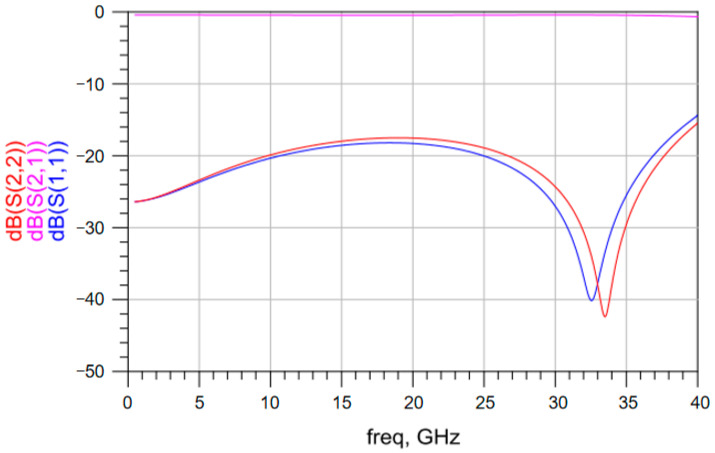
S parameters of the vertical interconnection structure based on solder balls with a diameter of 0.2 mm.

The S parameters of the vertical interconnection structure based on solder balls with different diameters were obtained. Figure 3 shows the simulated S parameters of the vertical interconnection structure based on solder balls with a diameter of 0.5 mm up to a frequency of 40 GHz. Above 20 GHz, the insertion loss changes significantly and the return loss exceeds −18 dB. Figure 4 shows the simulated S parameters of the vertical interconnection structure based on solder balls with a diameter of 0.3 mm up to a frequency of 40 GHz. The insertion loss of the vertical interconnection structure is better than −1 dB in the frequency range of 1–35 GHz. The return loss of the vertical interconnection structure is less than −18 dB. As shown in Figure 5, when the diameter of the solder ball is 0.2 mm, the insertion loss of the vertical interconnection structure is better than −1 dB in the frequency range of 1–40 GHz, and the return loss is less than −15 dB. This meets the transmission requirements of the vertical interconnection structure in the working frequency band.

These simulated results show that there is no significant difference in the insertion loss of the vertical interconnection structure based on solder balls with different diameters below a frequency of 20 GHz. In the high-frequency band, with the reduction of the solder ball diameter, the insertion loss and the return loss become smaller. This indicates that the vertical interconnection structure based on smaller solder balls provides better transmission performance.

### 2.2. Fabrication

In this section, the fabrication methods of the 1.5-level interconnections are shown in detail [26].

#### 2.2.1. Interconnection between Glass Micro Substrates

Ni/Au-plated copper balls were chosen for the interconnection between glass micro substrates. By utilizing non-collapsible copper balls, the actual interconnection length between the glass micro substrates is the same as the designed interconnection length.

The copper balls were connected to the substrate with use of Pb92.5Sn5Ag2.5 solder paste. For the copper core ball with a diameter of 0.5 mm, the mesh size of the solder mask was consistent with the size of the solder pad. Through experiments, the optimal range of solder paste thickness for preventing solder paste leakage was determined to be 0.12~0.15 mm. When the solder paste leakage thickness is less than 0.12 mm, insufficient solder at the solder joint and inadequate coverage of the copper core ball are likely to occur. Conversely, when the solder paste leakage thickness exceeds 0.15 mm, excessive solder can cause the copper core ball to be overcoated, affecting the interlayer stacking height, and even produce small solder beads and other excess materials.

Next, the reflow temperature and the welding temperature should be optimized. The liquidus temperature of Pb92.5Sn5Ag2.5 solder is 310 °C, and the welding temperature was set to 320 °C. The welding time range at this temperature was optimized. The optimized welding time range was 30~40 s.

#### 2.2.2. Interconnection between Glass Micro Substrates and Silicon Micro Substrates

For better RF performance, solder balls with diameters of ≤0.2 mm were used in the vertical interconnection between glass micro substrates and silicon micro substrates. Considering the RF performance and reliability of the vertical interconnection between different materials of micro substrates, SAC305 solder balls with a maximum diameter of 0.2 mm were selected.

Research was conducted on solder paste leakage parameters for alloy solder ball welding. The size of the solder paste leakage screen mesh was consistent with the size of the solder pad, and the leakage thickness was maintained at 0.05 mm.

For SAC305 solder balls, the melting point of the solder is 217 °C, and the welding temperature was set to 235 °C. At this welding temperature, the welding time range was optimized to 25~35 s.

#### 2.2.3. Interconnection between Silicon Micro Substrates and HTCC Substrates

Due to there being a much lower communication signal frequency between silicon micro substrates and HTCC substrates than that between micro substrates and silicon micro substrates, a larger diameter solder ball can be used in the vertical interconnection. Based on the electricity simulation results, a 0.3 mm diameter solder ball was employed in the interconnection. Considering the overall temperature gradient of the microsystem integration process, a Sn63Pb37 solder ball was selected.

For multi-layer stacked micro substrates, the cumulative alignment deviation caused by multiple stacking results in the deviation of the overall size of the micro substrates from the design value, which leads to a low yield of solder ball planting by traditional ball mounters. To address this problem, laser ball planting technology was adopted for solder ball mounting on stacked micro substrates.

For the Sn63Pb37 solder ball, both the reflow temperature and the welding temperature were optimized. The welding temperature was set at 210 °C. At this temperature, the welding time range was optimized to 30~40 s.

## 3. Results and Discussion

Based on the integration methods mentioned above, micro substrates with planted solder balls and interconnection samples of the micro substrates were manufactured. Then, the morphologies and phase structures of the solder balls in the interconnection structures were characterized by optical microscope, X-ray, and SEM.

**Figure 6:**
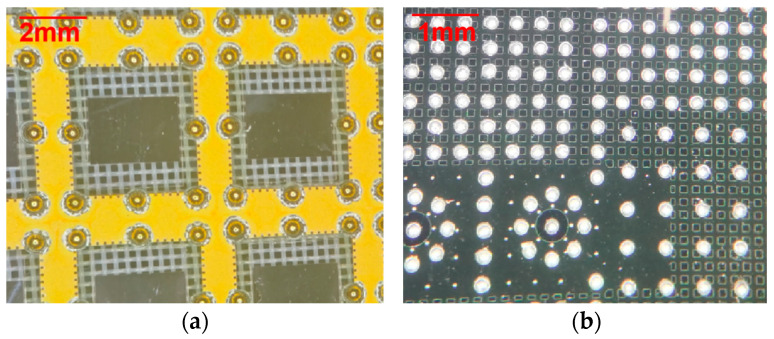
(**a**) The glass micro substrate with planted Ni/Au-plated copper balls and (**b**) the silicon micro substrate with mounted solder balls.

Figure 6a shows the glass micro substrate with planted Ni/Au-plated copper balls and Figure 6b shows the silicon micro substrate with mounted solder balls.

An image analyzer was used to measure ball grid array coplanarity, and the measured results are shown in Table 2.

It is evident that all the coplanarity values are less than 10% of the ball diameter, which benefits solder joint reliability.

Next, the shear force values of the solder balls mounted on the micro substrates were tested. Referring to the standard for solder ball shear (JESD22-B117, 2006), low-speed shear tests were implemented. The shear speed was 0.5 mm/s, and the shear tool stand-off was 0.08 mm. The test results are shown in Table 3.

The Shear force value is defined as

Shear force value (unit: N) = Shear force value (unit: kg) × g,

where g is the constant of acceleration due to gravity, g ≈ 10 m/s^2^. The shear force value (unit: kg) can be obtained from the shear testing.

The measured results show that the shear strength of the solder balls mounted on the micro substrates is sufficient for RF microsystem packaging.

**Figure 7:**
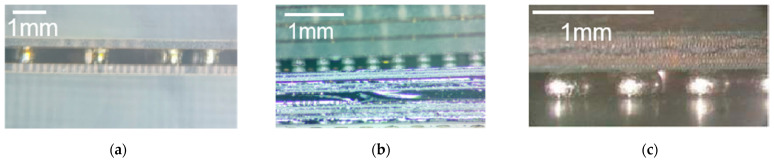
Microscope images of (**a**) the interconnection structure between glass micro substrates; (**b**) the interconnection structure between glass micro substrates and silicon micro substrates; and (**c**) the interconnection structure between silicon micro substrates and HTCC substrates.

Based on the integration methods and parameters outlined in Section 2, the micro substrate interconnection samples were manufactured and were subsequently investigated by microscope. As shown in Figure 7, excellent solder welding quality was obtained, characterized by a smooth surface, clear boundaries, and the absence of solder joint bridges.

**Figure 8:**
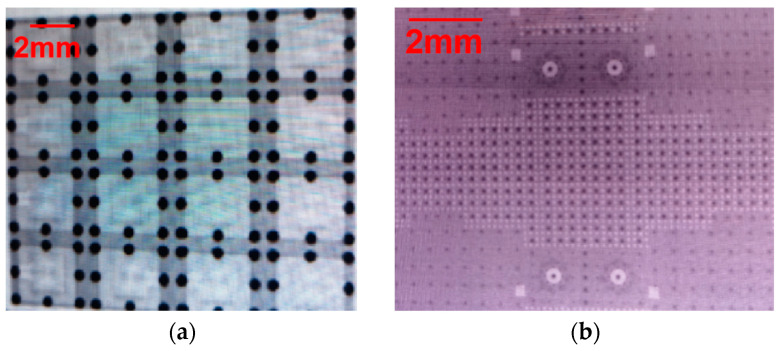
X-ray images of (**a**) an interconnection between glass micro substrates and (**b**) an interconnection between glass micro substrates and silicon micro substrates.

The micro substrate interconnection samples were investigated by X-ray. The X-ray images are shown in Figure 8. It is evident that there are no remainder particles in these figures. From these figures, we can determine that the void rate of the solder joint is less than 15%, and the solder ball offset is less than 25% of the solder pad diameter, which indicates good RF performances of the interconnection samples.

The micro substrate interconnection samples underwent and passed the Open and Short Electrical Test. Then, environmental stress screening of the solder joints was conducted using 500 temperature shocks at a 140 °C temperature difference. Subsequently, the microstructure, element composition, and distribution of the solder joints were analyzed by SEM EDS.

**Figure 9:**
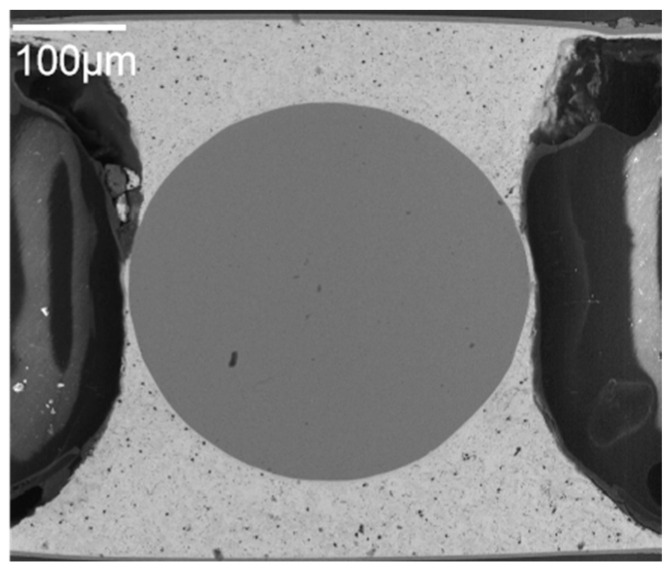
A SEM image of microstructure of Pb92.5Sn5Ag2.5 solder joint after 500 temperature shocks.

Figure 9 shows a SEM image of the microstructure of the Pb92.5Sn5Ag2.5 solder joint after 500 temperature shocks. There are no observable coarsened grains or microcracks in the solder joint.

**Figure 10:**
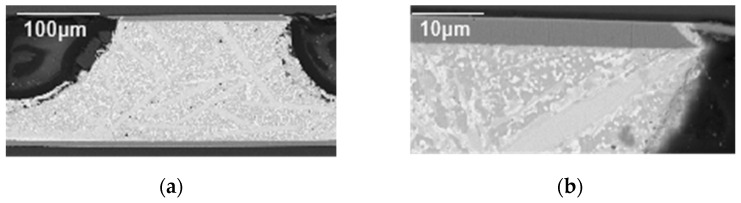
SEM images of microstructure of (**a**) SAC305 solder joint and (**b**) Sn63Pb37 solder joint after 500 temperature shocks.

As shown in Figure 10, no cavities, microcracks, or other kinds of defects were found in the SAC305 solder joint and Sn63Pb37 solder joint [27,28,29].

These measured results demonstrate that the interconnection structures manufactured based on the integration methods and parameters outlined in Section 2.2 exhibit high reliability.

## 4. Conclusions

In this paper, numerical models of 1.5-level interconnections were established using HFSS. The RF performances of 1.5-level interconnections based on solder balls with different diameters were analyzed. The optimized diameters of the solder balls applied in the vertical interconnections were obtained. Ni/Au-plated copper balls and Pb92.5Sn5Ag2.5 solder were selected for the vertical interconnections between glass micro substrates, ensuring the accuracy of the resonator length. SAC305 solder balls with a 0.2 mm diameter and Sn63Pb37 solder balls with a 0.3 mm diameter were selected for the interconnections between glass micro substrates and silicon micro substrates and between silicon micro substrates and HTCC substrates, respectively. SAC305 solder balls with a 0.2 mm diameter provided better RF performance in the high-frequency band. The integration process parameters of the vertical interconnections were optimized, and micro substrate interconnection samples were fabricated based on the optimized integration methods and parameters. The measured results of these samples demonstrate that the 1.5-level interconnections exhibit compatibility with the integration process and high reliability.

## Figures and Tables

**Table 1:** Material properties of the simulated structure.

Upper Layer/Lower Layer	Relative Dielectric Constant	Thickness (μm)
Glass/Silicon	3.3/11.9	180/180

**Table 2:** Measured results of solder ball array coplanarity (unit: mm).

Types of Solder Balls	Ni/Au-Plated Copper Balls	SAC305 Solder Balls	Sn63Pb37 Solder Balls
Ball diameter (mm)	0.5	0.2	0.3
Coplanarity value (mm)	0.03	0.02	0.02

**Table 3:** Shear strength of solder balls mounted on micro substrates.

Types of Solder Balls		Ni/Au-Plated Copper Balls	SAC305 Solder Balls	Sn63Pb37 Solder Balls
Ball Diameter (mm)		0.5	0.2	0.3
Shear force value (N)	#1	6.08	3.90	5.35
	#2	6.70	3.75	4.86
	#3	7.63	4.30	5.73
	#4	6.44	3.93	4.76
	#5	6.50	4.22	5.31
	Mean	6.67	4.02	5.20

## Data Availability

Data are available upon request from the authors.
